# The role of SASP in ischemic stroke: a deep dive into cellular mechanisms

**DOI:** 10.3389/fneur.2024.1513357

**Published:** 2025-01-27

**Authors:** Dong Xie, Yang Liu, Fang-Biao Xu, Jin-Sheng Zhang

**Affiliations:** ^1^The Third Clinical Medical College, Henan University of Chinese Medicine, Zhengzhou, China; ^2^Department of Encephalopathy, The Third Affiliated Hospital of Henan University of Chinese Medicine, Zhengzhou, China

**Keywords:** ischemic stroke, SASP, single cell sequencing analysis, immune microenvironment, basophils, cell communication

## Abstract

**Background:**

The escalating incidence of ischemic stroke (IS) exerts a heavy toll on global health. Aging, a prominent risk factor, implicates the senescence-associated secretory phenotype (SASP) in IS pathogenesis. We postulated that alterations in SASP-related factor expression during IS correlate with remodeling of intercellular interaction networks and disease advancement. The present study endeavored to preliminarily dissect the SASP-IS nexus via combined bulk and single-cell transcriptome analysis.

**Methods:**

Aggregated expression profiles from human peripheral blood bulk chips and MCAO mouse single-cell sequencing data, followed by SASP gene analysis. Executed protein interaction network and enrichment assays. Investigated immune infiltration in stroke patients, managed quality control and annotation of single-cell data, cherry-picked central cells based on SASP scores, unearthed essential genes via enrichment analysis, conducted pseudo-time and intercellular communication studies, and prognosticated drugs for hub genes. Finally, authenticated core gene expression in serum of MCAO and Sham rats using real-time fluorescent polymerase chain reaction (RT-qPCR).

**Results:**

Fourteen hub genes were discerned. Seven cell types were annotated in MCAO mouse peripheral blood single-cell data. Basophils exhibited the highest SASP scores, with Lcp1 upregulated and Ccl3 downregulated in basophils of the MCAO group. Enrichment analysis divulged a significant association of Ccl3 with the cell apoptosis pathway and Lcp1 with immune responses. The Ccl3 gene is pivotal in basophils and basophil-neutrophil crosstalk. Additionally, we forecasted nagrestipen’s regulatory function on Ccl3. RT-qPCR demonstrated a marked elevation in Lcp1 mRNA and a pronounced reduction in Ccl3 in the MCAO group relative to the Sham group.

**Conclusion:**

The Ccl3 gene in basophils and its immune cell interaction is a linchpin in the IS immune microenvironment. Ccl3 and Lcp1 might potentially modulate IS progression by influencing SASP, proffering novel prospects for IS clinical diagnosis and treatment.

## 1 Introduction

Ischemic stroke (IS) exhibits a serious health concern, as emphasized by a 2022 report from the American Heart Association, stating that roughly 795,000 people undergo strokes each year. About 610,000 of these cases are first-time incidents, with 185,000 recurrences (1). IS is the second leading death cause and stands as the third leading disability cause worldwide among adults (2). Notably, traditional risk factors like hypertension and diabetes do not completely elucidate every epidemiological and pathophysiological feature of all cases. Hence, it is vital to pinpoint more potential risk factors and therapeutic targets.

Aging emerges as a key risk factor for ischemic stroke (3, 4). Growing evidence has proven that aging significantly influences various neurovascular disease pathogenesis mechanisms (5, 6). SASP, a characteristic of aging cells, encompasses cellular factors such as pro-inflammatory cytokines, chemokines, and proteases that are omnipresent in aging cells. This pro-inflammatory phenotype exudes harmful cellular factors and other markers into the neighboring cell environment via paracrine signaling, providing the foundation of numerous age-related diseases (7–10). Nevertheless, the role of SASP factors within ischemic brain injury lesion areas or cell phenotypes remains partially understood.

Single-cell transcriptomics permit an in-depth understanding of individual cell gene expression patterns. This means revealing the disparities and diversity among cells, exploring their unique features and functions. Such technology considerably aids in understanding molecular mechanisms underlying disease conditions. It can assist in deciphering abnormal expression patterns in cells under disease conditions, thereby providing new insights for disease diagnosis and treatment (11, 12). There are still significant gaps and controversies in exploring post-stroke treatment targets using single-cell data. In existing studies, most focus on gene expression changes at the overall tissue level, and have not fully analyzed the heterogeneous responses of different cell subpopulations at the transcriptome level after stroke, especially how these changes interact and affect the overall neurological function recovery process. Currently, our understanding is quite limited.

**Figure 1:**
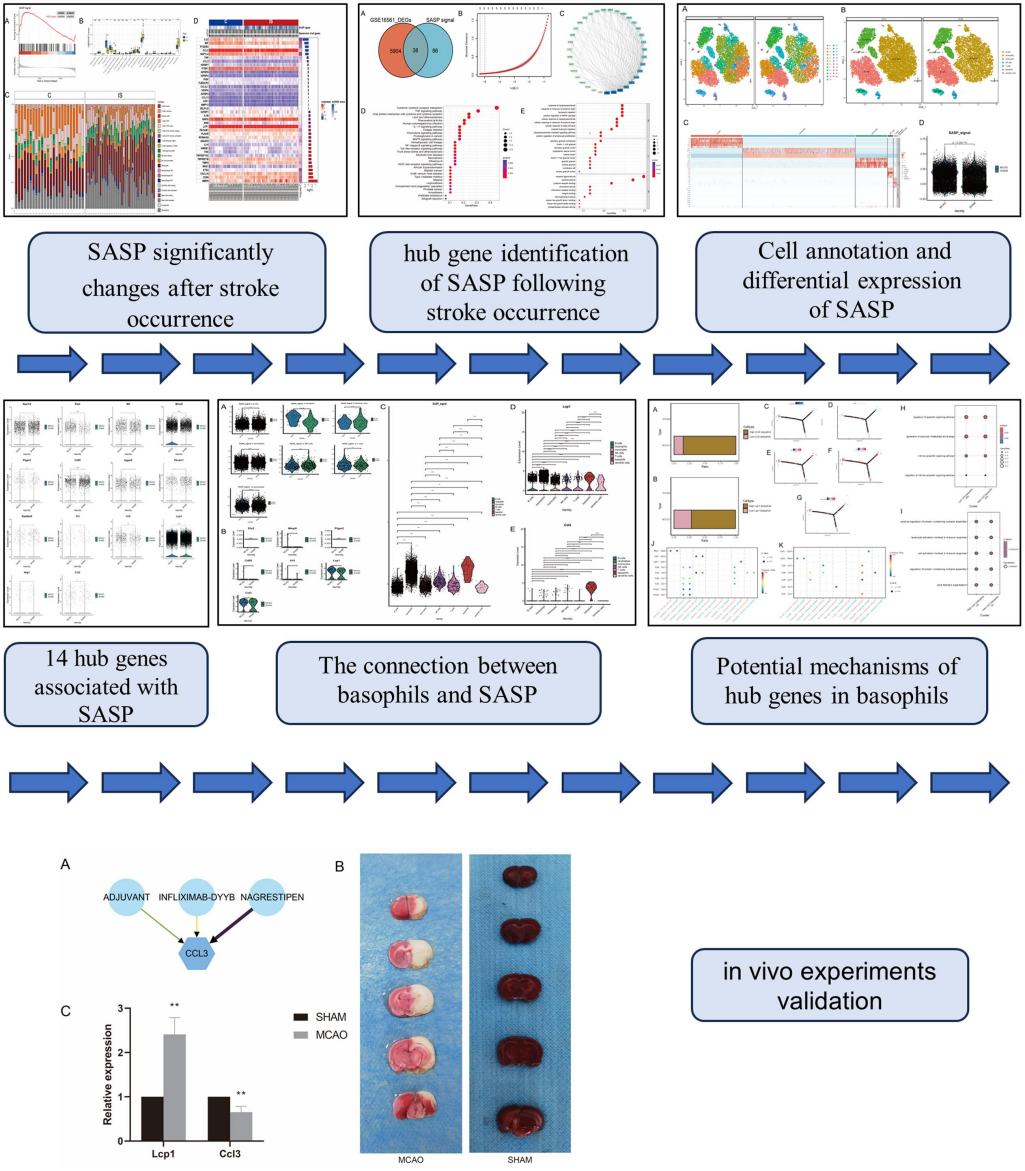
Flowchart.

Given the significant role of SASP in the production, progression, treatment, and prognosis of IS, this study combined state-of-the-art technology with combined bulk transcriptome and single-cell transcriptomic analysis. This approach aimed to explore deeply the link between SASP and IS, identify more potential risk factors and therapeutic targets, and furnish references for disease clinical diagnosis and treatment (Figure 1).

## 2 Methods

### 2.1 Data source and differentially expressed genes

The “ischemic stroke” keyword was used to search the GEO database and we procured the human peripheral blood bulk chip expression profile GSE16561, containing data from 39 ischemic stroke patients and 24 healthy controls. Additionally, we obtained a peripheral blood single-cell sequencing dataset GSE225948 from MCAO mice. The difference analysis was carried out on the chip data using the limma-trend (13) method. Probe sets without corresponding gene symbols were excluded, and genes with multiple probe sets were averaged. Genes with a *p*-value less than 0.05 and absolute log fold change equal or greater than 1 were declared as differentially expressed genes. The GSEABase R package (14) was utilized to conduct gene set enrichment analysis (GSEA) on the bulk dataset targeting the SASP gene set.

### 2.2 Immune infiltration analysis

Further analysis of the immune microenvironment in ischemic stroke patient’s peripheral blood was facilitated using the GSE16561 dataset. The immune cell infiltration condition was evaluated through the application of CIBERSORT (15) for deconvolving the expression matrix of human immune cell subtypes grounded on linear support vector regression principles. Differences in immune cells between ischemic stroke and the control group were assessed using the limma package.

### 2.3 Identification of hub genes in SASP

The VennDiagram R package (16) was used to analyze and visualize the overlaps between the differentially expressed genes in the bulk dataset and the SASP gene set. Overlapping genes were categorized as SASP-related genes. The protein interaction network of the SASP-related genes was retrieved from the STRING database (17) which provides comprehensive information about the protein interactions of queried gene sets. To reduce complexity, we performed the least absolute shrinkage and selection operator (LASSO) regression (18).

### 2.4 Enrichment analyses of SASP-related genes

The clusterProfiler R package (19) was employed to perform Gene Ontology (GO) and Kyoto Encyclopedia of Genes and Genomes (KEGG) enrichment analyses of SASP-related genes. KEGG, for instance, enables analysis of signal pathways potentially activated by the existing gene set, thus assisting in potential molecular mechanism elucidation.

### 2.5 Quality control of single-cell dataset

The single-cell data underwent a quality control check using the Seurat package. Samples were discarded if the detected feature count was less than 200, mitochondrial gene expression exceeded 10%, and ribosomal gene expression passed over 20%. After dimensionality reduction through PCA and batch effect elimination through Harmony, appropriate PCA count was selected based on PCA results. The UMAP was then used for secondary dimensionality reduction yielding a single-cell data matrix.

### 2.6 Manual annotation of single-cell data

Manual annotation was performed using the cell markers from the original dataset authors (20).

### 2.7 Differential expression of SASP gene set

We employed the Seurat package’s AddModuleScore function (21) to score SASP gene set, and the wilcox.cox test within the stat_compare_means function to perform differential analysis on individual cells and between MCAO and Sham.

### 2.8 Differential expression of hub genes

We used the ggpubr package’s stat_compare_means function (22) for Wilcoxon test to analyze the differential expression of hub genes in various cells and between MCAO and Sham. Based on changes observed between MCAO and Sham, and variance in hub genes expressions, a specific cell category was selected for further analysis.

### 2.9 Influence of hub genes in pivotal cells

For a more accurate understanding of activated signaling pathways due to varying hub gene expressions in key cells, our research divided cells into high expression and low expression groups based on median hub gene expression, to be followed by GO enrichment analysis.

### 2.10 Pseudo-time analysis

To delve deeper into the role of hub genes during pivotal cell differentiation, the monocle2 package (23) was utilized for pseudo-time analysis of key cells, with the grouping information of key genes being high and low.

### 2.11 CellChat

To better understand intercellular interactions, the CellChat package (24) was used to analyze the interplay between key cells and others.

### 2.12 Drug prediction of the hub genes

To predict drugs related to the hub genes, we utilized the DGIdb Database.^1^ This database integrates information from multiple sources, including literature curation, pharmacogenomic databases, and drug-target interaction data, providing a comprehensive resource for exploring potential drug candidates based on gene sets. All information was imported into the “Cytoscape” software to construct a network regulatory graph.

### 2.13 MCAO rat model creation and specimen collection

SPF grade Sprague Dawley rats (weighing 280–300 g) were used in the experiment, with animal certificate number 370726231100699867, provided by Jinan Pengyue Experimental Animal Breeding Co., Ltd., which was approved by the Ethics Committee of Henan University of Traditional Chinese Medicine (Approval No. IACUC-202305042). MCAO/R model (25) was used to simulate conditions in experimental rats, omitting cerebral artery wire occlusion block in the Sham group. Post 24-h modeling, a Longa Score (25) was performed with scores ranging from 1–3 chosen as the model group. Three animals each in the MCAO group and Sham group, at the end of a 7-day period of raising, blood from the abdominal aorta was extracted and TTC staining conducted on the brain.

### 2.14 Real-time fluorescent polymerase chain reaction

RNA was extracted from rat serum using the Trizol kit, with reverse transcription performed using the Takara kit. Real-time fluorescent polymerase chain reaction (RT-qPCR) involved setting up a reverse transcription system, executing RT-qPCR reaction, and calculating target gene mRNA expression levels.

### 2.15 Statistical analysis

Bioinformatics data analysis was conducted using R 4.2.3, and the Wilcoxon test was adopted for all comparisons of differences. The RT-qPCR data were analyzed using IBM SPSS Statistics 30 and a Student’s *t*-test was used for statistical comparisons. Statistics are deemed significant when ^*^p < 0.05, ^**^p < 0.01 or ^***^p < 0.001.

## 3 Results

### 3.1 Data sources and differentially expressed genes

**Figure 2:**
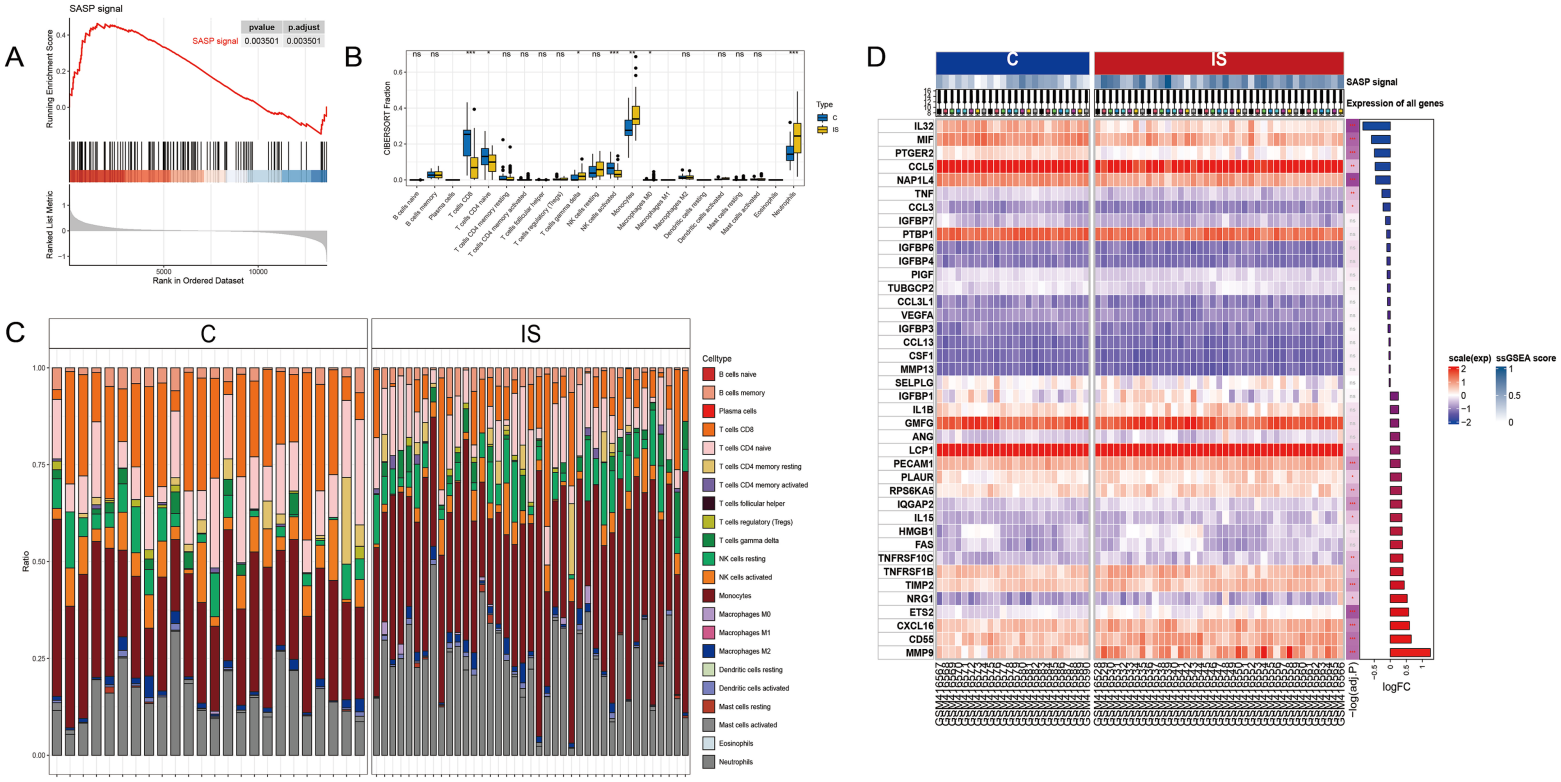
SASP significantly changes after stroke occurrence. **(A)** GSEA analysis after IS microarray data processing. **(B)** Box plots showing inter-group differences. **(C)** Relative content of 22 cell types in samples after CIBERSORT immune infiltration. **(D)** Top 20 genes with the most significant upregulation and downregulation fold changes.

Upon retrieval and selection, a total of 485 SASP-related genes were obtained. Post the processing of IS chip data, GSEA analysis was executed, the results of which are illustrated in Figure 2A. Comparing with the peripheral blood of healthy individuals, significant alterations were found within the SASP pathway in people with ischemic stroke. Figure 2D details 20 genes exhibiting the most significant upregulation and downregulation.

### 3.2 Immune infiltration analysis

A CIBERSORT immune infiltration analysis was conducted, resulting in the relative amount of 22 cells being represented in Figure 2C and the box plot of group differences in Figure 2B. The analysis indicated that CD8 T cell, CD4 naive T cell, and activated NK cells exhibited a significant decrease in the IS group, while gamma delta T cells, monocytes, and neutrophils cells experienced a significant increase in the IS group.

### 3.3 Identification of hub genes in SASP, LASSO regression and protein–protein interaction network construction

**Figure 3:**
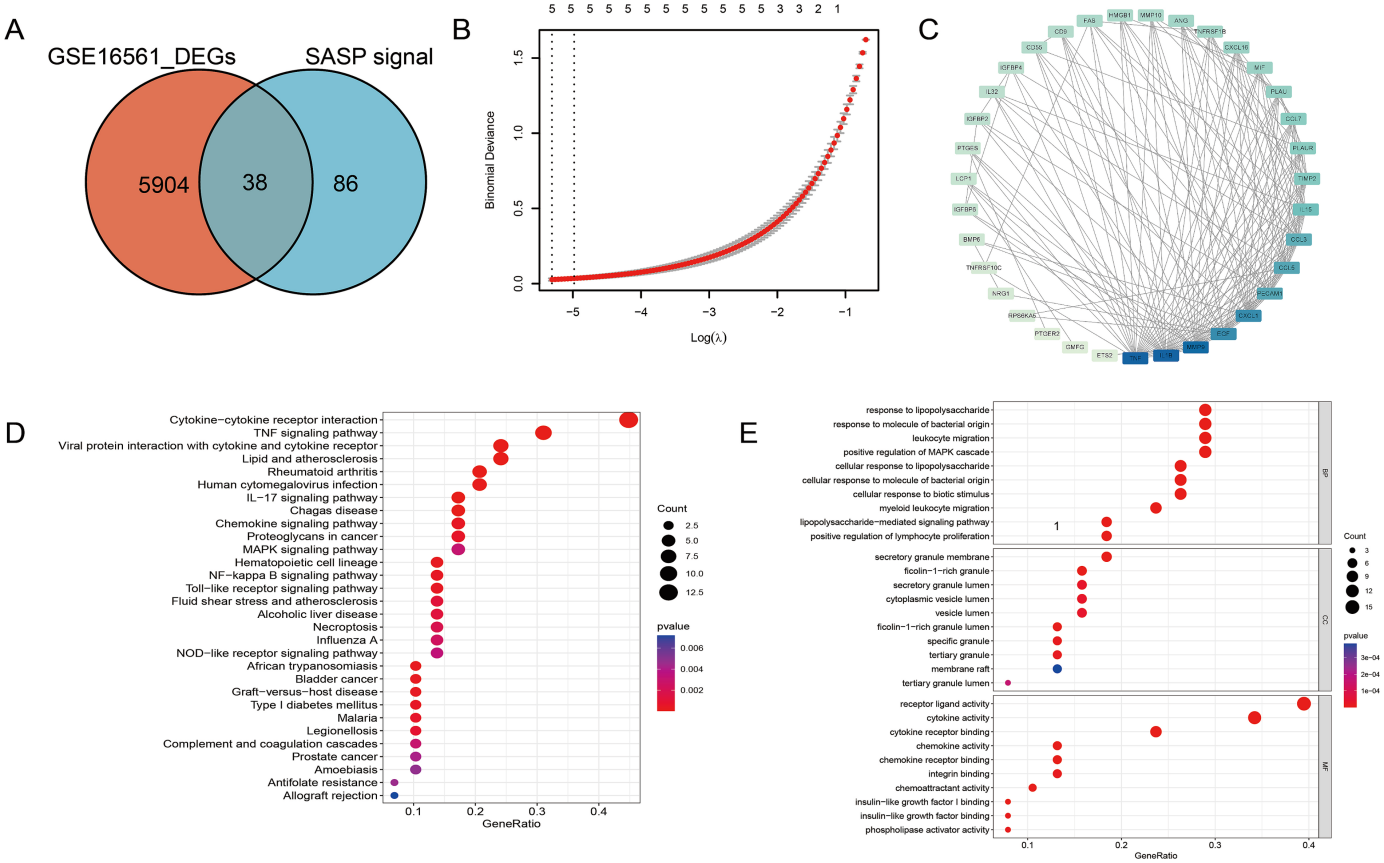
Hub gene identification of SASP following stroke occurrence. **(A)** SASP-related genes. **(B)** LASSO regression of SASP-related genes. **(C)** Protein interaction network among SASP-related genes. **(D)** The biological pathways and functional enrichments of SASP-related genes. **(E)** The specific molecular functions, cellular components and biological pathways related to SASP—related genes.

The presence of SASP could exacerbate the neuroinflammatory response, impacting the function of neuroglial cells and the blood-brain barrier, as well as possibly impeding neuroprotection and repair. To gain a better understanding of the gene association between SASP and ischemic stroke, we conducted a differential analysis on the human peripheral blood bulk chip expression profile GSE16561, a total of 5,904 differential genes were identified, 38 of which were consistent with the SASP pathway and hence marked as SASP-related genes (Figure 3A). SASP-related genes were introduced into the STRING database to obtain their protein interactions. Module analysis, biological process enrichment, and LASSO regression were conducted subsequently (Figures 3B,C). From the enrichment results, we identified 14 hub genes that are associated with SASP and ischemic stroke for further evaluation.

### 3.4 Enrichment analyses of SASP-related genes

To discern the biological processes and pathways involved in SASP-related genes, both GO and KEGG enrichment analyses were executed (Figures 3D,E). It is noteworthy that within the KEGG enrichment, the cytokine–cytokine receptor interaction pathway and the NF-κB pathway exhibited prominence. From the results, it is clear that SASP has a significant association with the interaction between bacteria-derived molecules and leukocytes.

### 3.5 Single-cell dataset analysis

**Figure 4:**
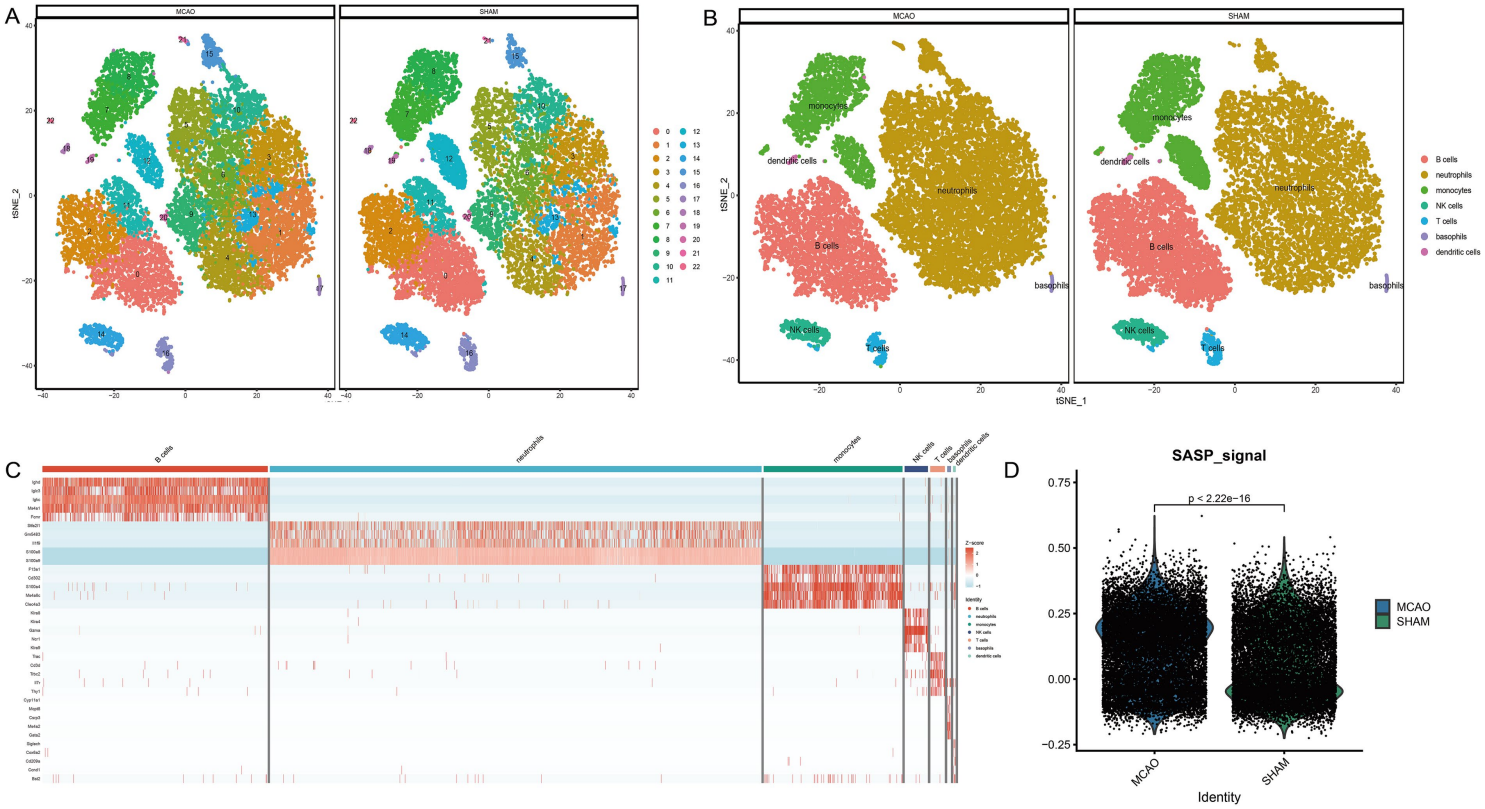
Cell annotation and differential expression of SASP. **(A)** Clustering of different cell types in the single-cell sequencing dataset of mouse peripheral blood. **(B)** Cell annotation of different cell types in the single-cell sequencing dataset of mouse peripheral blood. **(C)** Differential expression analysis between cell types and between MCAO and Sham groups. **(D)** SASP scores in MCAO and Sham groups.

The single-cell sequencing dataset GSE225948 of MCAO mouse peripheral blood was obtained, followed by quality control and dimensionality reductions. The Umap presentation and the final annotated Umap image are depicted in Figures 4A,B, respectively. Resultantly, seven cell types were annotated, with neutrophils being the most represented (Figure 4C). The scoring of SASP for both the MCAO and Sham groups can be seen in Figure 4D. As indicated by the results, SASP underwent a significant transformation after the onset of ischemic stroke.

### 3.6 Single-cell dataset verification of key gene expression differences

**Figure 5:**
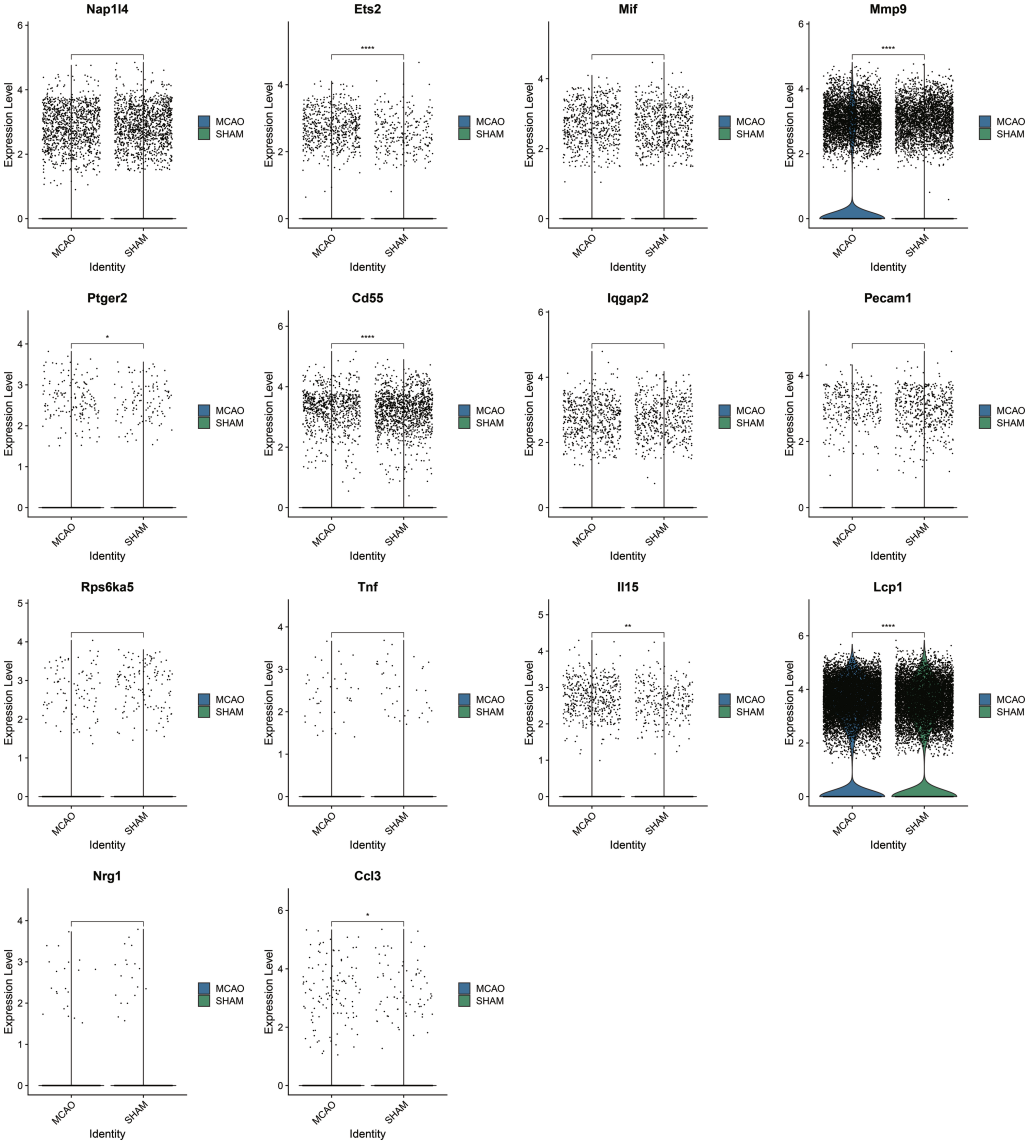
Fourteen hub genes associated with SASP.

Upon selecting 14 key SASP-associated genes, only seven genes demonstrated differences between the MCAO and Sham groups within the single-cell dataset (Figure 5). They include Ccl3, Lcp1, Il15, Cd55, Ptger2, Mmp9, and Ets2, with particularly significant differences in the genes Ccl3 and Lcp1. As these seven genes underwent cross-validation in both bulk and single-cell datasets, they are believed to play a vital role in SASP pathological alterations.

### 3.7 Analysis of SASP changes from a cellular perspective

**Figure 6:**
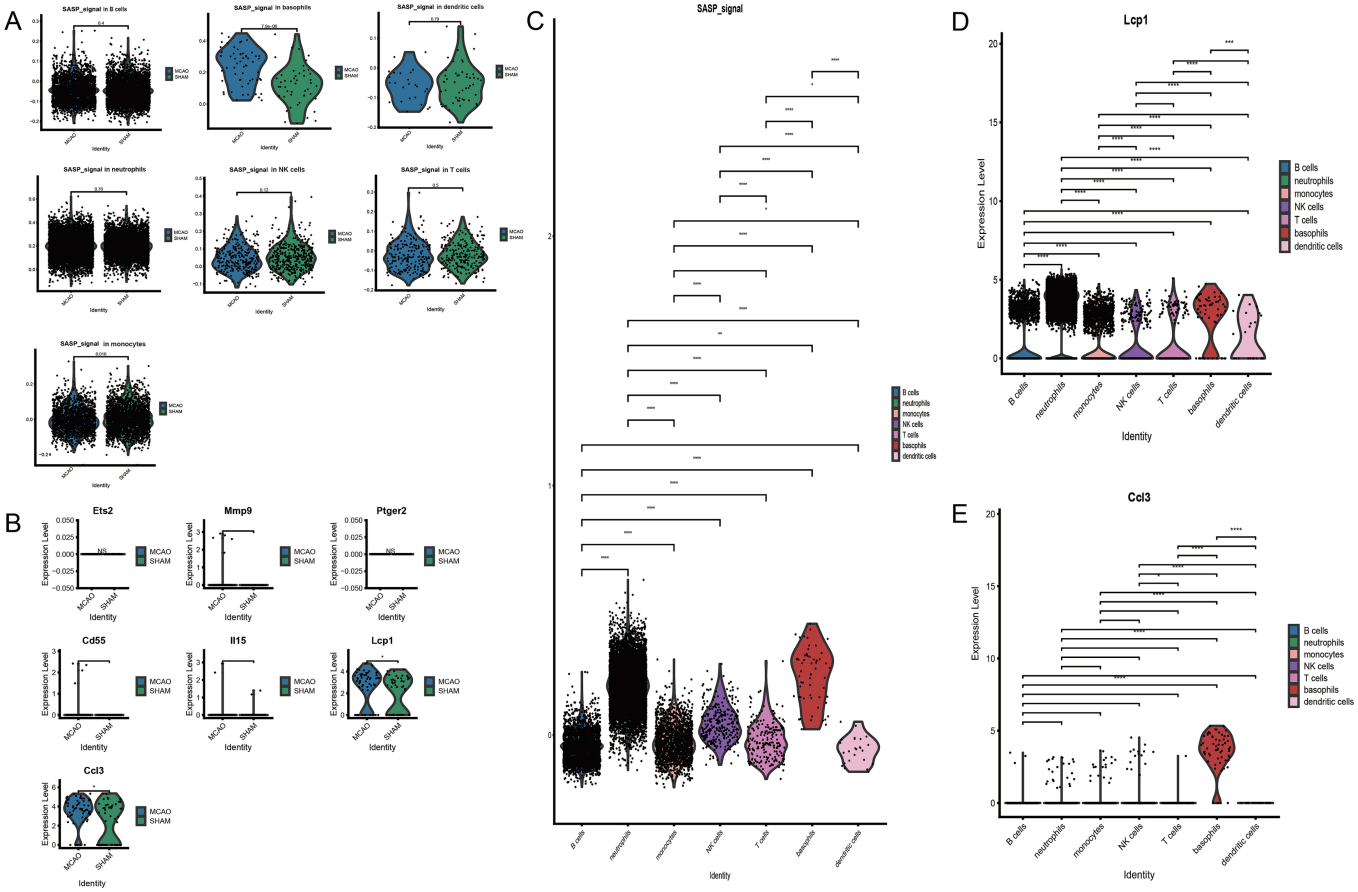
The connection between basophils and SASP. **(A)** Inter-group comparison of SASP differences in various cells. **(B)** Differential expression of key SASP genes in basophils. **(C)** SASP expression differences in various cells following stroke. **(D)** Differential expression of Lcp1 genes in various cells. **(E)** Differential expression of Ccl3 genes in various cells.

To understand the activation level of SASP post the onset of ischemic stroke from a cellular viewpoint, SASP scores for each cell type were extracted and intergroup comparisons were performed between MCAO and Sham and between different cells (Figure 6A). Significant differences were noted in basophils cells between the MCAO and Sham groups. When compared pairwise among cells in the MCAO group, SASP was prominently represented in basophils and neutrophils. Despite basophils having the highest SASP score, the highest number of occurrences was witnessed in neutrophils. However, this does not necessarily suggest that SASP is highest in neutrophils. Through comparison, we concluded that the most significant change within SASP post the onset of ischemic stroke occurred in basophils. Therefore, subsequent focus will be on basophils to continue exploring SASP’s key genes, differentiation state of basophils, and interactions between basophils and other cells.

### 3.8 Investigating key genes of SASP changes in basophils

Post cross-validation with a bulk dataset and a single-cell dataset, seven key genes of SASP were established. Their expression differences in basophils were further validated, with the results indicated in Figure 6B. Only two genes, Ccl3 and Lcp1, were found to have significantly elevated expression in the MCAO group, with a statistically significant difference when compared with the Sham group. As indicated by the figure, basophils majorly expressed Ccl3, while all cells to varying extents expressed Lcp1. To illustrate in detail the differential expression of SASP signal and hub genes in various cells, violin plots were plotted for pairwise comparison (Figures 6C,D,E). The figure evidently shows that in the MCAO group, Lcp1 is expressed in B cells, neutrophils, monocytes, NK cells, T cells, basophils, dendritic cells, but a greater expression is concentrated in neutrophils and basophils. Ccl3 is primarily expressed in basophils. Both the Ccl3 and Lcp1 genes exhibit certain cell specificity and are worth additional comprehensive study.

### 3.9 Single gene analysis

**Figure 7:**
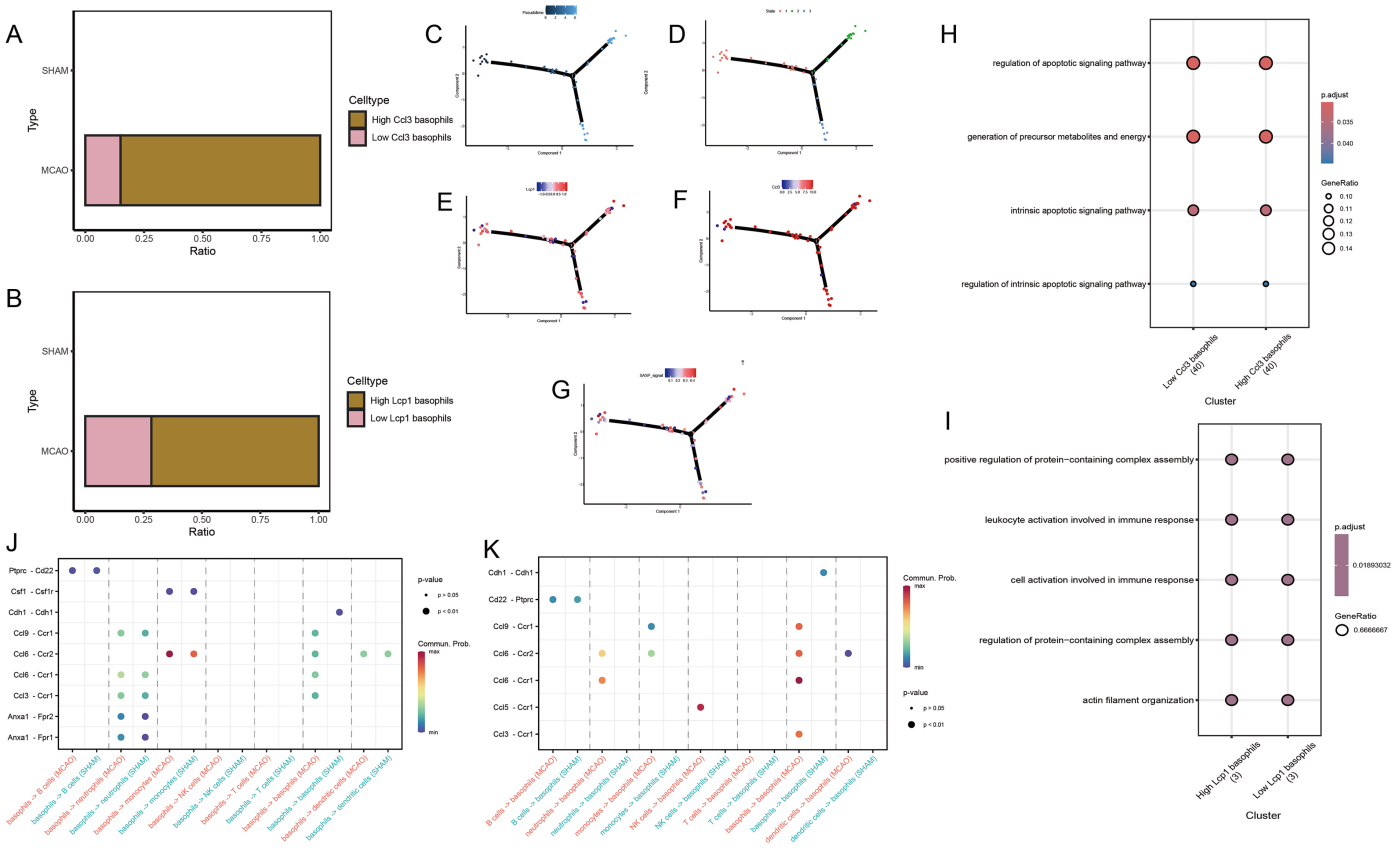
Potential mechanisms of hub genes in basophils. **(A)** High Ccl3 basophils group and low Ccl3 basophils group based on median Ccl3 gene expression. **(B)** High Lcp1 basophils group and low Lcp1 basophils group based on median Lcp1 gene expression. **(C,D)** Differentiation status of basophils. **(E)** Change in Lcp1 expression along the pseudo-time developmental tree. **(F)** Change in Ccl3 expression along the pseudo-time developmental tree. **(G)** Change in SASP signal along the pseudo-time developmental tree. **(J,K)** Cell communication study between MCAO and Sham groups. **(H)** The specific molecular functions, cellular components and biological pathways of differentially expressed genes between high and low Ccl3 expression groups in basophils. **(I)** The specific molecular functions, cellular components and biological pathway of differentially expressed genes between high and low Lcp1 expression groups in basophils.

To better comprehend the phenotypic changes that Ccl3 and Lcp1 genes may cause in basophils, we extracted basophils from the MCAO group. Based on the median expression of Ccl3 and Lcp1 genes, we divided them into high Ccl3 basophils group and low Ccl3 basophils group, high Lcp1 basophils group and low Lcp1 basophils group. The grouping situation is represented in Figures 7A,B. Further GO enrichment analysis revealed that the expression of Ccl3-related pathways in basophils significantly relates to the cell apoptosis pathway (Figure 7H) while the Lcp1-related pathway has a significant association with the immune response (Figure 7I).

### 3.10 Pseudo-time analysis

Monocytes were extracted for pseudo-time analysis to better understand the differentiation status of monocytes. The pseudo-time development tree, depicted in Figures 7C,D, reflects determined branch points and staging. Subsequently, the expression changes of Ccl3 and Lcp1 genes were evaluated in the pseudo-time development tree (Figures 7E,F,G). From both figures, it is apparent that in the early stages of basophils, cells with high expression of Ccl3 and Lcp1 are relatively abundant, while they are relatively scarce at the terminal stage.

### 3.11 CellChat

To conduct cell communication studies between the MCAO group and the Sham group, all cell types were utilized, with basophils acting as both receptor and source cells (Figures 7J,K). A significant difference was noted in the ptprc-cd22 pathway. Interestingly, the Ccl3-Ccr1 pathway also appeared in these differing pathways. Analysis found that the Ccl3-Ccr1 pathway is not only a critical pathway linking basophils as source cells and neutrophils as receptor cells, but the activation level of this pathway in the Sham group is lower than that in the MCAO group. In cell communication with basophils serving as both source cells and receptor cells, the Ccl3-Ccr1 pathway also shows significant expression, and the activation level of this pathway in the Sham group is lower than that in the MCAO group.

### 3.12 Drug prediction of the hub genes

**Figure 8:**
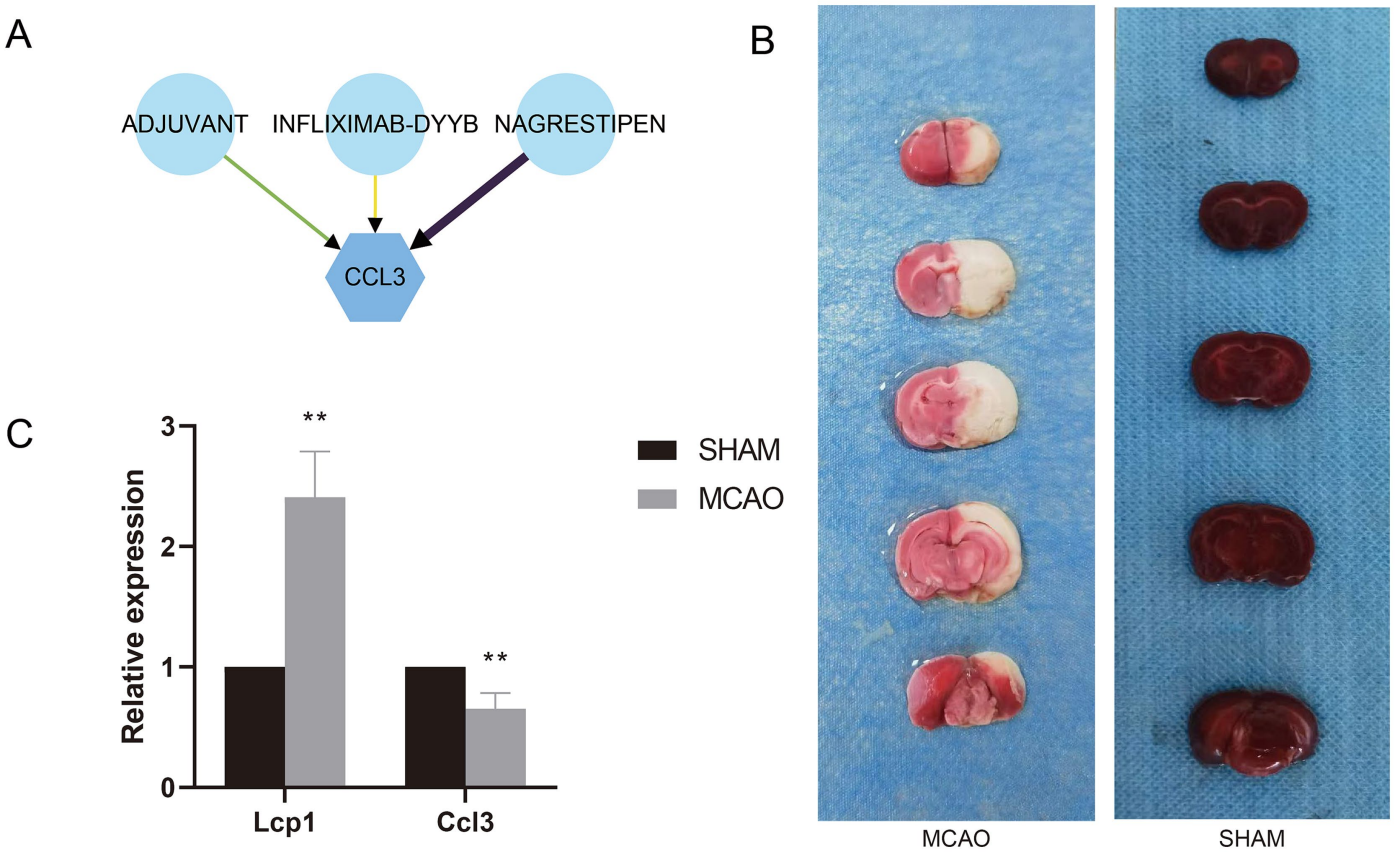
**(A)** Drug-gene interaction network. **(B)** TTC staining of MCAO and Sham groups. **(C)** mRNA expression levels of hub genes in MCAO and Sham groups.

A total of three small drug molecules that have regulatory effects on Ccl3 were screened out in the DGIdb database, namely ADJUVANT, INFLIXMAB-DYYB, and NAGRESTIPEN. Among them, NAGRESTIPEN exhibits a higher degree of reliability (Figure 8A).

### 3.13 Validation of expression levels of prognostic signature genes

The rat brain infarction area was evaluated by TTC staining (Figure 8B). Compared with the Sham group, the rat brain infarction area in the MCAO group significantly increased, proving successful modeling. Aligning with previous research results, compared with the Sham group, the mRNA expression of the Lcp1 gene in the serum of the MCAO group rats significantly escalated, while the mRNA expression of the Ccl3 gene significantly decreased (Figure 8C).

## 4 Discussion

Ischemic strokes are induced by obstructions within blood vessels that transport oxygen and nutrients to the brain, resulting in tissue homeostasis and neurological function disruption. Ischemia reperfusion (IR) triggers cellular phenomena including oxidative stress and neuroinflammation, which lead to cell damage. Aging stands a primary risk factor for ischemic stroke (3, 4). Ongoing research implies substantial aging role in neurovascular disease pathogenesis, with annual risk and severity growth from the age of 55 (5–7). Despite effective ischemic stroke therapies reducing mortality rates, post-stroke morbidity is mostly unaltered. Recent insights highlight the contribution of cellular aging and senescence-associated secretory phenotype (SASP) to brain injury and neurodegenerative alterations. SASP, ubiquitous in aging cells, originates via various processes (cellular damage, DNA damage, oncogenic transformation, inflammation, oxidative stress), subsequently establishing a pro-inflammatory phenotype. This phenotype results in adverse cell factors and marking substance excretion into surrounding cellular environments via paracrine signaling. These excretions form many age-related diseases’ groundwork (8–10). SASP and aging involvement in inflammation is imperative; here, pro-inflammatory cytokines in synch with immune cells instigate low-grade sterile chronic inflammation, categorically termed “inflammation” (4, 26). Several inflammation-inducing systems like IL-6, IN-1a, TNF-α, and NLRP3 are SASP constituents that amplify aging pathology and inflammation, worsening SASP effects on neighboring cells (5, 27, 28). Besides chronic low-intensity sterile inflammation, active inflammation initiated by damaging cell injury and natural aging influence the NF-kB inflammation pathway associated with SASP. Varied cellular injuries induce a cellular aging state characterized as resistance to apoptosis enhancement, cell cycle stagnation, and SASP increment (29). Numerous studies underline aging and SASP roles in ischemic stroke pathogenesis, proposing them as potential therapeutic targets.

Considering SASP’s pivotal post-ischemic stroke role, we administered GO and KEGG enrichment analyses of biological procedures and signaling channels comprising SASP-related genes. Of importance in KEGG enrichment is the selection of the cytokine–cytokine receptor interaction and NF-κB pathways. Complete cellular aging is when cells transcriptionally reshape and totally withdraw from the cell cycle, during which SASP aging markers and phenotypic modifications occur (30, 31). Various growth agents, cytokines and proteases secrete into the extracellular space. Among these, factors such as IL-1α and IL-6 set up self-secretory pro-aging feedback systems and transmit signals to adjacent cells via paracrine. Among these elements, the most universally adopted mechanism is the NF-kB signaling pathway, which induces pro-inflammatory cytokines IL-6 and IL-8 (SASP’s principal constituents) with remarkable expression (9, 30, 32). From the BP enrichment results, strong SASP association with bacterium-derived molecule interaction with leukocytes is evident. The leukocytes, upon bacterium-derived molecule recognition (like lipopolysaccharides), activate a variety of immune responses leading to cellular stress and damage, hence inducing aging, culminating in the SASP exhibitance, and potentially disrupting immune system equilibrium and encouraging persistent inflammation that further enhances cellular aging and SASP development.

Post-ischemic stroke SASP changes primarily occurred within basophil cells. Subsequent extraction of SASP scores of individual cell types and separate SASP scoring for MCAO and Sham groups revealed notable SASP alterations post-ischemic stroke. Further exploration showed that post-ischemic stroke SASP changes primarily occurred within basophil cells. Following ischemic stroke, damaged brain tissue expels damage-associated molecular patterns (DAMPs), which stimulate immune cells, triggering inflammatory reactions. Basophil granulocytes perceive these signals and activate, initiating inflammatory mediators while recruiting immune cells like neutrophils, monocytes, and macrophages to the damaged area. These cells subsequently discharge inflammatory mediators and reactive oxygen species, thereby intensifying tissue damage.

More profound surveys of basophils’ effect on SASP enabled the extraction of two crucial genes (Ccl3 and Lcp1) within basophil cells’ significant impact on SASP. Ccl3’s influence over SASP regulation within the apoptosis pathway inside basophil cells. Meanwhile, post-stroke, we posit that basophils in the front line undergo pathological alterations and, through the Ccl3-Ccr1 pathway, guide neutrophils and unaffected basophils to effect chemotactic function, participate in the removal of pathological cells and tissue repair. Noteworthy is the chemokine (C-C motif) ligand (Ccl) family that can recruit and activate immune cells while also adjusting the types of immune responses. Ccl3’s influence over SASP regulation within the apoptosis pathway inside basophil cells was discovered in our recent investigation. Following stroke onset, blood supply to the brain is interrupted, precipitating local tissue ischemia and hypoxia. This instigates a surge of pathological physiological changes including energy metabolism disruption, oxidative stress, inflammatory responses, excitotoxicity, calcium overloading, and mitochondrial dysfunction, which activate and intensify cell apoptosis, hence, boosting the loss of neurological function. After categorizing basophil cells based on Ccl3 expression and performing differential analysis, apoptosis-related pathways were found to be significantly enriched. This suggests potential Ccl3 involvement in post-stroke basophil cell apoptosis. Concurrently, as a pivotal player in SASP phenotypic changes, Ccl3 plays a crucial role in SASP’s effects on apoptosis. Existing studies have found (33) that SASP can heighten cell apoptosis by activating inflammatory responses, such as the NF-κB and JNK pathways. Conversely, the TGF-β growth inhibitory factor’s activation in SASP can boost cell apoptosis—consistent with our findings. From our findings, we can reasonably hypothesize a significant activation of SASP phenotypes following a stroke, potentially prompting basophil apoptosis. Ccl3 appears to play a pivotal role in this progression, possibly serving as a primary therapeutic target for post-stroke treatment. Our research excitingly revealed enhanced interactions between basophils and neutrophils via the Ccl3-Ccr1 pathway post-stroke, compared to the Sham group. Interestingly, we observed the emergence of the Ccl3-Ccr1 pathway in basophil self-interactions after stroke. CCR1, a G protein-coupled receptor predominantly expressed in immune cells, is located on the cellular surface—specifically, the cell membrane. By bonding with particular chemokines, such as CCL3, CCL4, CCL5, CCR1 facilitates cell chemotaxis, directing immune cell migration towards inflammatory or infection sites, a function vital for initiating and modulating immune responses (34). Post-stroke, we posit that basophils in the front line undergo pathological alterations and, through the Ccl3-Ccr1 pathway, guide neutrophils and unaffected basophils to effect chemotactic function, participate in the removal of pathological cells and tissue repair. These findings underscore the critical role of Ccl3 in basophils, suggesting its potential as a key post-stroke therapeutic target.

Lymphocyte cytosolic protein 1 (LCP1) plays a fundamental role in the immune response. Lcp1, another gene significantly impacting the basophil SASP, emerged through our research, appearing to primarily influence basophil immune response. The immune response following an ischemic stroke is indeed complex; initially, brain ischemia, hypoxia, and tissue injury cause the release of various damage associated molecular patterns (DAMPs) from damaged brain cells. Recognized by pattern recognition receptors (PRRs) on immune cell surfaces, these DAMPs instigate an immune response, mobilizing immune cells towards damaged brain tissue. In response to DAMPs and other stimuli, these immune cells activate, further exacerbating the inflammatory response through the release of pro-inflammatory cytokines, chemokines, reactive oxygen species, and proteases. LCP1 plays a fundamental role in the immune response, especially integral to the oxidative burst and signal transduction process. As a crucial component of the NADPH oxidase complex, responsible for generating superoxide and other reactive oxygen species (ROS), such as hydrogen peroxide (H_2_O_2_) and hypochlorous acid (HOCl), it assists in eradicating pathogens and damaged cells. Moreover, LCP1 features in various signal transduction pathways, including the MAPK and NF-κB pathways, the activation of which enhances cytokine and chemokine expression, thereby boosting the immune response (35). These findings intimate that Lcp1 likely participates in the immune response sequence following a stroke, potentially influencing the stroke’s onset and progression.

Although some studies have preliminarily identified some genes or signaling pathways that may play a key role after stroke, the dynamic expression patterns of these potential targets at single-cell resolution and their precise correspondence with cellular functional phenotypes have not been clearly elucidated. The differences in single-cell sequencing technology platforms, sample processing methods, and data analysis processes used in different studies have led to poor reproducibility and comparability of reported potential therapeutic targets, sparking controversy over the reliability and universality of certain targets. For example, Zhao et al. (36) and Fu et al. (37) applied partially overlapping gene sets for single-cell data analysis, but the results obtained different hub genes and immune cells containing these hub genes, which may be due to differences in experimental technique details and analysis methods.

Our research found that there is a previously unrecognized role and dynamic expression between Ccl3 and Lcp1 in basophils in the context of ischemic stroke, providing a new perspective for the synthesis of pathophysiological networks. Future research can focus on translating these findings into clinical applications. However, this study also has certain limitations. For instance, the conclusions drawn herein mainly rely on bioinformatics analysis, and there might be some deviations in the single-cell data. In the experimental verification part, the sample size of animal models is relatively small, and no gene knockout and overexpression experiments were conducted for further validation. Therefore, although this study offers a new perspective on the pathophysiological network of IS, their clinical relevance remains speculative. Substantial efforts and further investigations are still required to bridge the gap between the findings of this study and their practical application in the human clinical realm. Furthermore, it is necessary to further investigate the role of SASP in different subtypes of ischemic stroke and comorbidities patient populations, which may contribute to personalized treatment strategies.

## 5 Conclusion

Advancements in large-scale single-cell analyses and cell communication research have substantially enriched our understanding of SASP’s role in ischemic strokes. Specifically, Ccl3 gene expression in basophils and its interaction with immune cells appears to significantly shape the immune microenvironment of IS. Furthermore, Ccl3 and Lcp1 genes in basophils seemingly influence IS progression by impacting SASP.

## Data Availability

The original contributions presented in the study are publicly available. This data can be found here: https://www.ncbi.nlm.nih.gov with accession numbers GSE16561 and GSE225948, further inquiries can be directed to the corresponding authors.

## Footnotes

^1^ https://dgidb.org/
